# A Chromosomal-Level Genome of *Dermatophagoides farinae*, a Common Allergenic Mite Species

**DOI:** 10.1155/2024/3779688

**Published:** 2024-04-24

**Authors:** Rongxuan Hu, Haifeng Huang, Ying Zhou, Yanshan Liu, Yaning Ren, Yuanfen Liao, Cunyin Yuan, Xiaohong Gu, Yubao Cui

**Affiliations:** ^1^Department of Rheumatology, The Affiliated Children's Hospital of Jiangnan University, Wuxi 214023, China; ^2^Dermatological Department, The Affiliated Wuxi People's Hospital of Nanjing Medical University, Wuxi 214023, China; ^3^Department of Pediatrics Laboratory, The Affiliated Children's Hospital of Jiangnan University, Wuxi 214023, China; ^4^Clinical Research Center, The Affiliated Wuxi People's Hospital of Nanjing Medical University, Wuxi 214023, China; ^5^Respiratory Department, The Affiliated Children's Hospital of Jiangnan University, Wuxi 214023, China

## Abstract

**Background:**

Genome data have been used to find novel allergen from house dust mites. Here, we aim to construct a chromosome-level genome assembly of *Dermatophagoides farinae*, a common allergenic mite species.

**Methods:**

We achieved a chromosome-level assembly of *D. farinae*'s genome by integrating PacBio single-molecule real-time sequencing, Illumina paired-end sequencing, and Hi-C technology, followed by annotating allergens and mapping them to specific chromosomes.

**Results:**

A 62.43 Mb genome was assembled with a 0.52% heterozygosity rate and a 36.11 Merqury-estimated quality value. The assembled genome represents 92.1% completeness benchmarking universal single-copy orthologs with a scaffold N50 value of 7.11 Mb. Hi-C scaffolding of the genome resulted in construction of 10 pseudochromosomes. The genome comprises 13.01% (7.66 Mb) repetitive sequences and predicts 10,709 protein-coding genes, 96.57% of which are functionally annotated. Moreover, we identified and located 36 allergen groups on specific chromosomes, including allergens Der f 1, Der f 2, Der f 23, Der f 4, Der f 5, Der f 7, and Der f 21 located on chromosomes 2, 1, 7, 3, 4, 6, and 4, respectively.

**Conclusion:**

This comprehensive genomic data provides valuable insights into mite biology and evolutionary adaptations, potentially advancing *D. farinae* allergy research and treatment strategies.

## 1. Introduction

*Dermatophagoides farinae* (*D. farinae*) and *Dermatophagoides pteronyssinus* (*D. pteronyssinus*) are domestic mite species commonly inducing atopic sensitization and allergy including affecting the eyes, upper and lower airways, skin, and, sometimes, systemic circulation [1]. More than 20% of people are affected by house dust mites (HDM) worldwide, and 30% of them carry asymptomatic HDM sensitization [2]. A cross-sectional survey of 6304 patients with asthma, rhinitis, or both in 17 cities from 4 regions of China revealed that the overall prevalence of positive skin prick responses was 59.0% for *D. farinae* and 57.6% for *D. pteronyssinus* [3].

An organism that induces allergy is defined as an allergen source, whereas an allergenic molecule (i.e., allergen component) is considered a molecule (i.e., protein or glycoprotein) derived from an allergen source identified using specific IgE antibodies. Therefore, allergen molecules are isolated from a natural allergen source (i.e., native, purified allergens) or can be produced with recombinant DNA technology [4]. Since HDM are allergen sources, their allergen components have been investigated since the 1980s to explore methods for personalized diagnosis and treatment. In total, 39 allergen groups of IgE-binding components with similar sequences among different mites have been identified from these *Dermatophagoides* species and recorded by the World Health Organization (WHO) and International Union of Immunological Societies (IUIS) Allergen Nomenclature Sub-Committee, including 36 groups in *D. farinae* and 30 in *D. pteronyssinus* [5].

In recent years, genome assemblies of allergenic mites have been an essential resource for finding novel allergen groups through homology search of various nonmite sources. The first mite genome of *D. farinae* was reported in 2015, which contained the full gene structures of 20 canonical allergens and 7 noncanonical allergen homologs; a major allergen, ubiquinol-cytochrome c reductase-binding protein-like protein, was identified and designated as Der f 24 [6]. In 2017, *de novo* draft genome assembly method was used for *D. pteronyssinus*; Der p 16, Der p 22, and Der p 25 till Der p 35 were identified as novel allergen groups [7]. In 2018, *D. pteronyssinus* genome was generated from a population of randomly breeding diploid dust mites that had been maintained as a colony for several years; allergen isoforms and variations were examined at the genome level in this unique case [8]. These genome data obtained through high-throughput DNA sequencing expedite novel allergen identification. However, the above-mentioned mite genomes [6–8] were assembled from short-read DNA sequences, resulting in gaps in the genomes and no information regarding the linkage groups, which has impeded the understanding of the house dust mite genome structure.

In 2021, Chen et al. extracted genetic material from *D. farinae* bodies and eggs and sequenced with short reads from next-generation sequencing (NGS) and long reads from PacBio/nanopore sequencing; compared with the *D. farinae* draft genome, genome size was corrected (from 53.55 Mb to 58.77 Mb), and the contig N50 was increased (from 8.54 kb to 9365.49 kb). The assembled genome has 10 contigs, 33 canonical allergens, and 2 novel allergens (Der f 37 and Der f 39) [9].

High-throughput/resolution chromosome conformation capture (Hi-C) is frequently used to provide links across various length scales, even spanning entire chromosomes. In contrast to paired-end reads from cloned libraries, any given Hi-C contact spans an unknown length and may connect loci from different chromosomes [10]. In recent years, Hi-C has been used to improve draft genome assemblies and create chromosome-length scaffolds for large genomes [11–14]. In the present study, we generated the chromosome-level genome of *D. farinae* through long-read Pacific Bioscience sequencing, Illumina paired-end sequencing, and Hi-C-based chromatin contact maps; subsequently, the allergens of *D. farinae* were annotated and assigned to relevant chromosomes. Accordingly, we created a chromosome-level assembly of *D. farinae* to serve as the most comprehensive genomic data available for an allergenic mite species.

## 2. Methods

### 2.1. Mite Culture and Purity Evaluation

*D. farinae* mites were cultured in our laboratory for many generations in cell culture flasks (75 cm; Corning, USA) in an artificial climate incubator (RXZ-280D; Jiangnan Instrument Factory, Ningbo, China) at 25°C ± 1°C and 85% ± 5% relative humidity [15]. *D. farinae* was identified during cultivation through the observation of its morphological characteristics. To protect the integrity of the DNA, live mites (~3,000) were collected by hand, washed with 75% ethanol, and immediately frozen and stored in liquid nitrogen.

### 2.2. Mitochondrial Genome Sequencing

A purity check of the *D. farinae* mites was performed through complete mitochondrial genome sequencing. Total genomic DNA was extracted using a TIANamp Micro DNA Kit (Tiangen Biotech, Beijing, China). After random shearing using a Covairs ultrasonic breaker, we fragmented DNA for genomic DNA library construction by using the whole-genome shotgun strategy. The DNA fragments were repaired at their protruding ends by using a combination of 3′-5′ exonuclease and polymerase, and a single “A” base was introduced at the 3′ end of the segment for fragment ligation; this promoted selective enrichment of DNA fragments with joints at both ends while amplifying the DNA library. DNA quality was determined through 1% agarose gel electrophoresis and analyzed on an Agilent 2100 Bioanalyzer (Agilent Technologies, Santa Clara, CA, USA). DNA quantity was detected on a NanoDrop 2000 spectrophotometer (Thermo Fisher Scientific, Waltham, MA, USA). Subsequent sequencing was performed on an Illumina NovaSeq platform in the paired-end 150 bp sequencing mode. De novo construction of mitochondrial sequences was performed using A5-miseq v2015052239 [16] and SPAdesv3.9.040 [17], followed by the alignment of the high-depth sequencing results with the mt library of the NCBI database by using BLAST (version 2.2.31) [18]. We then used MUMmer (version 3.1) [19] for collinearity analysis to determine the positional relationships between different contig sequences. The complete mitogenome sequence was revised and corrected using Pilon (version 1.1842) [20] and then uploaded to the MITOS web server [21] for functional annotation under default settings and the genetic code 02-Invertebrate.

### 2.3. *D. farinae* Genome Sequencing

The Illumina HiSeq X Ten platform (Illumina, San Diego, CA, USA) and the PacBio Sequel platform (Pacific Bioscience, San Diego, CA, USA) were used to perform genome sequencing to generate short and long reads, respectively. A paired-end Illumina genomic library was generated and sequenced according to the Illumina protocol. For long-read sequencing on the PacBio Sequel sequencer, a 20 kb single-molecule real-time sequencing bell library was constructed using a PacBio DNA Template Prep Kit 1.0. The quality and quantity of each library were assessed using the Agilent 2100 Bioanalyzer (Agilent Technologies). Templates were selected according to size, and long fragments (>10 kb) were enriched for sequencing using BluePippin (Sage Science, Inc., Beverly, MA, USA).

### 2.4. Genome Survey, Initial Genome Assembly, and Quality Evaluation

#### 2.4.1. K-mer Analysis and Genome Feature Estimation with Illumina Sequencing Data

Raw data from Illumina paired-end reads were processed using fastp (version 0.20.1) [22]. Adaptors reads with low-quality bases and reads containing >10% unknown bases (“*N*”) were removed from the analysis. Subsequently, k-mer frequency distribution analysis was performed, as described previously [12]. The k-mer was set to 17 for further analysis. “kmer_freq_stat” [23] was used to estimate genome size, the presence of repetitive sequences, and genome heterozygosity. The formula for genome size estimation was as follows: *G* = *N* k − mer/*D*, where *N* k − mer is the total number of k-mers, *D* is the peak depth of the k-mer, and *G* is the size of the genome. The histogram of the k-mers was generated using Jellyfish (version 2.3.0) [24], and GenomeScope (version 1.0) [25] was used to evaluate genomic characteristics.

#### 2.4.2. Genome Assembly and Chromosome Construction through Long-Read Sequencing

For genome assembly, we first used the open-source FALCON and FALCON-Unzip algorithms Falcon [26], WTDBG2 [27], and Flye [28] to assemble high-quality PacBio subreads independently. The homologous contigs were further optimized and corrected based on self-alignment and sequencing depth. The reads assembled using Falcon, WTDBG2, and Flye were merged using Quickmerge [29]. The merged genome was corrected with the Illumina data using NextPolish and Pilon [30].

The assembled genome quality was evaluated by mapping the short reads from Illumina to the assembly by using BWA-MEM (version 0.7.10-r789) [31], BUSCO (version 3.0.2) in addition to the arachnida_odb10 database, and Merqury (version 1.1) with special parameters (*k* = 21).

### 2.5. High-Throughput Chromosome Conformation Capture Sequencing and Chromosome Assembly

DNA fixation, chromatin isolation, and library construction were performed to construct a Hi-C chromatin contact map for chromosome-level assembly, according to the manufacturer's instructions [32]. First, the purified mite bodies were washed twice with phosphate-buffered saline (pH 7.4) and homogenized into a powder by using a mortar and pestle with liquid nitrogen. The homogenized mite samples were then incubated at 50°C for proteolysis, digested with the restriction enzyme *Hind*III, labeled with biotinylated nucleotides, and end repaired. After the reversal of the crosslinks, the ligated DNA was purified and sheared to a length of 300–700 bp. Biotinylated DNA fragments were captured with streptavidin beads and used for Hi-C fragment library construction. Finally, the quality and quantity of the purified library were verified using a Qubit 3.0 fluorometer (Invitrogen), Agilent Bioanalyzer 12-kb DNA Chip (Agilent Technologies), and quantitative polymerase chain reaction (PCR). A high-quality Hi-C fragment library was prepared for the Illumina HiSeq 2500 platform.

Sequencing data from the Hi-C library were used for chromosome-level assembly [33]. To obtain uniquely mapped read pairs, the raw data were aligned with the initial genome assembly by using BWA-MEM (version 0.7.10-r789) [34]. HiC-Pro [35] was used to evaluate valid Hi-C data based on uniquely mapped read pairs. Only valid read pairs were used for draft genome recorrection and chromosome-level genome assembly. The contigs of the draft genome were split into simulated 500 kb contigs, and LACHESIS [33] was used to sort these contigs into groups with the following parameters: CLUSTER_MIN_RE_SITE = 29, CLUSTER_MAX_LINK_ DENSITY = 2, CLUSTER_MONINFORMATIVE_RATIO = 2, ORDER_MIN_N_ RES_ TRUN = 15, and ORD-ER_MIN_N_RES_I-N-SHREDS = 15. Potential contamination by other organisms was removed during mounting; the subreads were compared with the deep sequencing results by using Bowtie2 and SAMtools. A Hi-C visualization heatmap was created using HiCPlotter, and a chromosome diagram was drawn using Circos. Heatmap colors ranging from light yellow to dark red were used to indicate the frequency (from low to high) of Hi-C interactions [35].

### 2.6. RNA Extraction, Library Construction, Transcriptome Sequencing, and Read Processing

Total RNA was extracted using a Qiagen 74104 Rneasy Mini Kit (Qiagen), according to the manufacturer's protocol. Total RNA quantity and purity were analyzed on a Bioanalyzer 2100 and RNA 6000 Nano LabChip Kit (Agilent, CA, USA). Next-generation sequencing libraries of transcriptomes were constructed using the SMART cDNA Library Construction Kit (CLONTECH Company, code no. 634901). mRNA was enriched and purified with magnetic beads (oligo (dT)) and then fragmented into small pieces by using divalent cations at high temperatures; these cleaved mRNA fragments were randomly sheared and used as templates to produce cDNA. This cDNA was purified, its sticky ends were restored to flat ends, A-bases were added to the 3′ ends, and joints were added as well. Finally, PCR amplification was performed. The library was submitted for quality control on the Agilent 2100 Bioanalyzer and ABI StepOnePlus Real-Time PCR system and then sequenced on the Illumina HiSeq 2000 instrument to produce paired-end 2 × 100 bp reads, according to the vendor's recommended protocol. Sequencing data were pretreated, assembled, and annotated as described in our previous publications [36, 37].

### 2.7. Repetitive Sequence Analysis and Genome Annotation

Repetitive elements were annotated based on homology and ab initio. LTR_FINDER (RRID: SCR_015247) (Benson), RepeatScout (RRID: SCR_014653) [38], and RepeatModeler (RRID: SCR_015027) were used to construct an ab initio repetitive element database, and RepeatMasker (RRID: SCR_012954) [38] was used to annotate repetitive elements on the basis of the database. Subsequently, RepeatMasker and RepeatProteinMask [39] were used to search the genome sequence for known repetitive elements, with the genome sequences used as queries against the Repbase database [40]. Tandem repeats were predicted using the Tandem Repeats Finder (Benson). We masked the repetitive regions of the assembled genome sequences by using RepeatMasker (version 4.0.5) [39].

For protein-coding gene prediction, we used both *de novo* and homology-based strategies in accordance with the MAKER pipeline [38]. Ab initio gene prediction was performed on the repeat-masked genome assembly by using Genscan, GlimmerHMM (version 3.0.4), GeneID (version 1.4), SNAP (version 2006-07-28) [41], and AUGUSTUS (version 2.4) [42]. For homology-based predictions, Hisat (version 2.0.4), StringTie (version 1.2.3), GeneMarkS-T (version 5.1), and PASA (version 2.0.2) were used, and EVM (version 1.1.1) was used to integrate the four sets of results. Finally, we mapped the allergen sequences onto the mite chromosomes. The homology alignment of protein-coding genes was performed using the public protein databases Swiss-Prot, TreEMBL, and Pfam by using BLASTX, with an *E-*value of 10^−5^.

### 2.8. Allergen Identification

We downloaded the sequences of the known allergen groups of *D. farinae* from the WHO/IUIS Allergen Nomenclature Sub-Committee website and searched for them in our genome. The protein database was created using BlastP with an *E*-value cutoff of 1*e*-50. Most match sequences in our genome met two standards: (1) having the highest score during BlastP and (2) being a top hit in hmmsearch with an HMM model constructed from known allergens downloaded from the WHO/IUIS website. When the HMM model was not used, the most matched sequences were the target during BlastP. When *D. farina*e allergen groups were unavailable (i.e., no reported allergen groups of *D. farinae* were present), we searched the entire Arthropod data to identify potential novel allergen groups. To confirm our genome annotation further, each identified candidate allergen was aligned with sequences deposited on the WHO/IUIS website.

## 3. Results and Discussion

### 3.1. Mitochondrial Genome Structure Confirmed High Purity of *D. farinae* in Our Study

**Figure 1:**
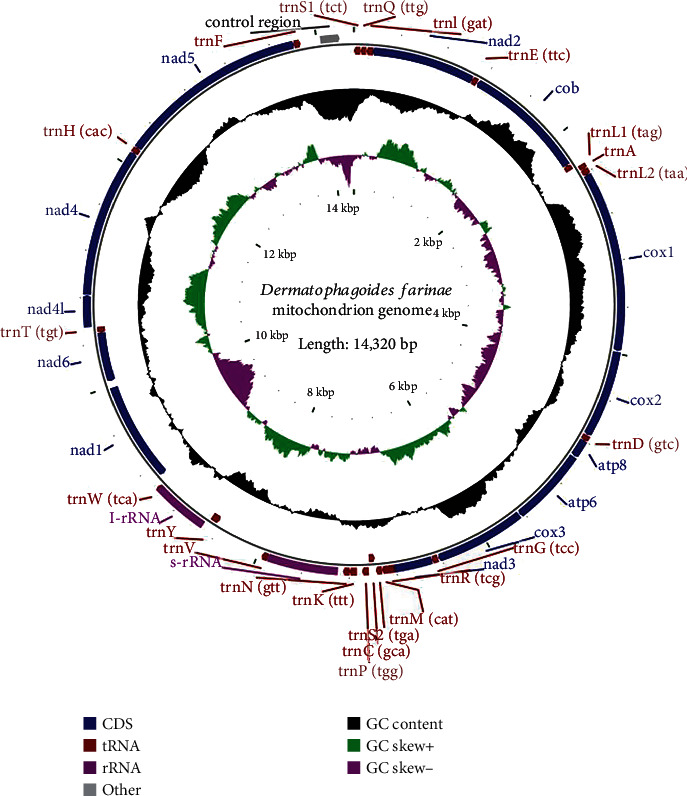
Schematic of the mt genome of *Dermatophagoides farinae*. Sequence coding for amino acids in proteins, transfer RNAs (tRNAs), ribosomal RNAs (rRNAs), and others is marked in blue, red, purple, and gray, respectively. tRNA genes are abbreviated using one-letter amino acid codes, and anticodon sequences are listed in parentheses. The nucleotide distribution is represented with *GC* content and the *GC* skew index. The *GC* skew index was calculated using the formula (*G* − *C*)/(*G* + *C*).

The final assembled mitochondrial genome of *D. farinae* had a length of 14,320 bp and included 13 coding genes, 22 transfer RNA genes, 2 ribosomal RNA genes, and 1 control region (Figure 1), which was submitted to the National Center for Biotechnology Information (NCBI) database under the accession number OR197578. The length and arrangement of the mitochondrial genome from our study were similar to the previously reported mitochondrial genome of *D. farinae*, which has a length of 14,266 bp (GenBank: GQ465336.1), suggesting the high purity of *D. farinae* used in our research.

### 3.2. Initial Genome Assembly and Evaluation

**Figure 2:**
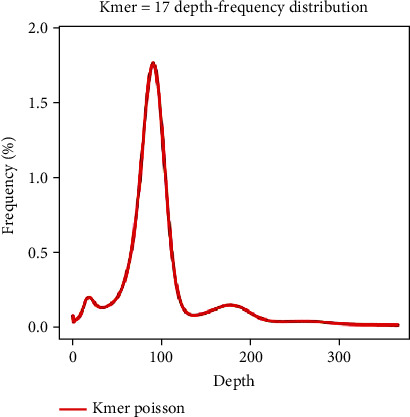
17-mer count distribution for *Dermatophagoides farinae* genome size estimation. Observed 17-mer count distribution for genome size estimation. Note that the peaks around the depths of 45, 89, and 178 represent the heterozygous, homozygous, and repeated k-mers, respectively.

Illumina platform generated 6.53 Gb data from a 400 bp insert library, representing a 105-fold coverage of the *D. farinae* genome. K-mers (*K* = 17) were extracted from a paired-end library with an insert size of 400 bp. We calculated and plotted the 17-mer depth distribution (Figure 2). The 17-kmer distribution showed a major peak at 89× (Figure 2). Based on the number of k-mers and relative k-mer depth, we estimated the genome size of *D. farinae* to be 59.35 Mb, according to the following formula: Genome size = kmer_number/Peak_depth.

PacBio platform generated 8.21 Gb high-quality sequences from the long-read library with a mean subread length of 7442 bp and read N50 value of 9679 bp, representing a 131-fold coverage of genome assembly. These PacBio reads resulted in an assembly with a contig N50 value of 273,396 bp.

### 3.3. Genome Quality Evaluation and Final Genome Assembly

The assembled genome was analyzed using benchmarking universal single-copy orthologs (BUSCOs; version 5.3.2) with the arachnida_odb10 database to assess its completeness. Of the 2934 total BUSCO groups searched, 2667 complete and single-copy ortholog groups and 35 complete and duplicated ortholog groups were identified. The assessment results indicated a relatively complete genome.

Considering the presence of symbiotic microorganisms in the digestive tract of mites, it is impossible to obtain pure mite bodies as sequencing samples [6, 7, 43–45]. Thus, we manually removed the microbial DNA contamination during genome assembly. Briefly, by conducting BLAST searches against the microbial RefSeq database with a stringent cutoff (*E*‐value ≤ 1*e*^−50^), the mite sequences were distinguished and separated from microbiota sequences. After filtering, we obtained a final genome size of 62.43 Mb from the Hi-C library. A previous minireview reported that the genome sizes of *D. farinae*, *D. pteronyssinus*, *Euroglyphus maynei*, *Blomia tropicalis*, and *Tyrophagus putrescentiae* were 63.7 Mb, 66.6 Mb, 43.4 Mb, 62.7 Mb, and 97.4 Mb, respectively [46], ranging from 43 to 100 Mb, which is inclusive of our genome size.

The National Center for Biotechnology Information (NCBI) database contains three *D. farinae* genomes: (1) GCA_000767015.2 [47], with a total length of 60.39 Mb and a scaffold N50 value of 8.98 Mb; (2) GCA_002085665.2 [48], with a total ungapped length of 91.9 Mb and a scaffold N50 value of 0.19 Mb; and (3) GCA_020809275.1 [9], with a total length of 58.8 Mb and a Contig N50 value of 9.3 Mb. Due to PacBio technology, this scaffold-level genome assembly includes 10 contigs scaffolds [9], but no assembled chromosomes. These *D. farinae* genome data were downloaded from NCBI, reassembled and annotated, and compared with our current findings (Table 1). Our genome has a total length of 62.43 Mb and an N50 value of 7.12 Mb. In genome assemblies, N50 is the measure of assembled genome quality. The higher the N50 value is, the higher the quality of the corresponding genome assembly becomes [49]. By using Merqury (version 1.1) [50], we found the assembly consensus quality value of our genome to be 36.1083, representing an accuracy of 99.975%. Therefore, the *D. farinae* genome presented in our study is of high quality.

**Figure 3:**
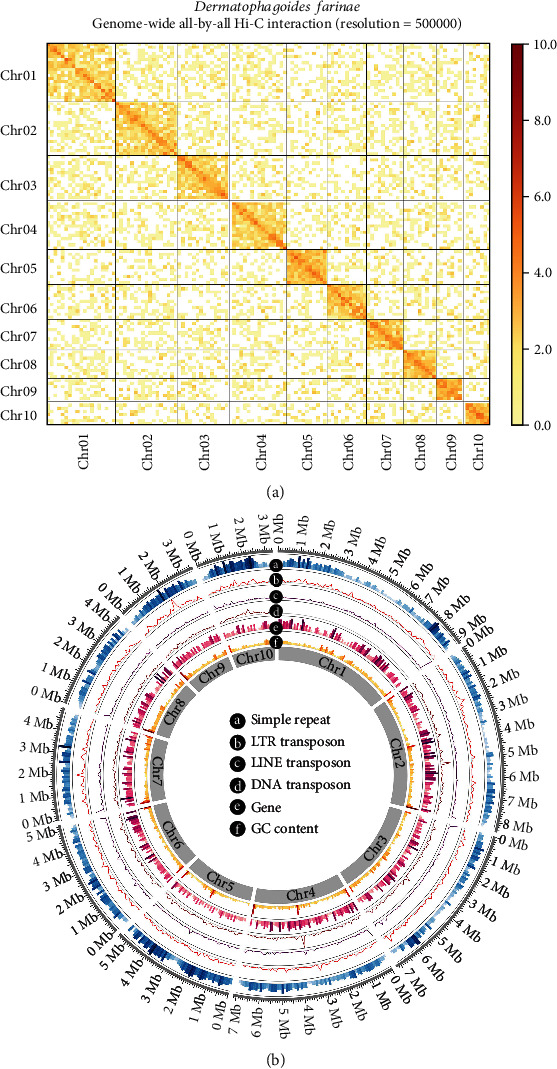
Features of the *Dermatophagoides farinae* genome. (a) Genome-wide high-throughput chromosome conformation capture (Hi-C) map with a 500 kb resolution. Strong interactions are indicated in bright yellow, whereas weak interactions are indicated in light yellow. Chr denotes chromosome and represents 10 pseudochromosomes. The blocks represent contact between one location and other locations. The color bar displays the contact density from low (yellow) to high (red). (b) Circos plot of 10 chromosome-level scaffolds, representing the annotation results of genes, ncRNAs, and transposable elements on these scaffolds.

Finally, 62.43 Mb of data from 21 scaffolds were anchored onto and oriented toward 10 pseudochromosomes through agglomerative hierarchical clustering. A heatmap of the interactions among the pseudochromosomes illustrated that genome assembly was complete and robust (Figure 3(a)). The sizes of the 10 pseudochromosomes ranged from 9,172,953 to 3,348,946 bp (Figure 3(b)).

### 3.4. Gene Annotation

After integrating several programs and results and eliminating redundancy, we estimated that 13.01% (7.66 Mb) of the *D. farinae* genome comprised repeat sequences (Table 2). Of the classified repetitive elements, simple repetitive elements were determined to be the most abundant (11.41%) in the genome.

In total, 10,709 protein-coding genes were identified in the *D. farinae* genome. The total coding sequence length was 17,338,048 bp, with an average transcript length of 1619.02 bp. Moreover, the total number of mRNA sequences was 100,538, with an average length of 793.03 bp.

In total, 10,709 genes, representing 96.57% of the predicted genes, were efficiently annotated with their putative functions (Table 3). With our functional annotation analysis, 9524 (88.93%), 6487 (60.58%), and 9853 (92.01%) genes demonstrated significant hits with proteins cataloged in the InterPro, Swissprot, and NR databases, respectively (Table 3). Moreover, 7612 (71.08%), 5000 (46.69%), 5930 (55.37%), and 7853 (73.33%) genes were annotated in the Pfam, KEGG, GO, and eggNOG databases, respectively (Table 3).

### 3.5. Allergen Identification and Location

**Figure 4:**
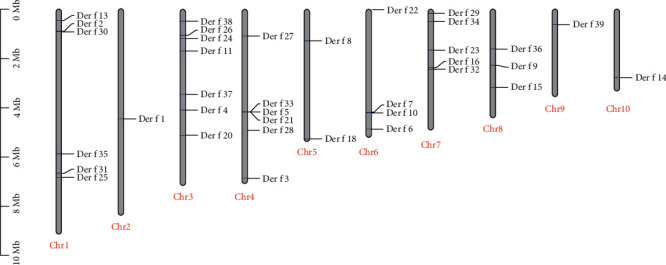
Loci of identified allergen genes on 10 chromosomes. All known *Dermatophagoides farinae* allergens in IUIS are shown in approximate locations of our assembled 10 chromosomes (Chr), and the imaginary lines connect the physical locations.

We identified 36 allergen groups, and our sequences were determined to exhibit an excellent match with known allergen groups deposited in IUIS, with percent identities ranging from 83.99% to 100% for nucleotide sequences (Table 4). However, we did not find Der f 12, Der f 17, and Der f 19 and assigned these allergen groups to each chromosome of *D. farinae* on the basis of our chromosome assembly results (Figure 4). The *Dermatophagoides* allergens were classified into four categories according to their IgE-binding frequency: serodominant (groups 1, 2, and 23), midtier (groups 4, 5, 7, and 21), minor (groups 3, 6, 8, 9, 10, 11, 13, 15, 16, 17, 18, and 20), and allergen of unknown importance [51]. The major allergens Der f 1, Der f 2, and Der f 23 are located on chromosomes 2, 1, and 7, respectively. The midtier allergens Der f 4, Der f 5, Der f 7, and Der f 21 are located on chromosomes 3, 4, 6, and 4, respectively. Der f 24, an allergen group identified from the first *D. farinae* genome [6], is located on chromosome 3. Allergens of groups 3, 6, and 9 allergens are serine protease trypsin, chymotrypsin, and a collagenolytic enzyme, respectively [52, 53]. The present study located Der f 3 and Der f 6 on chromosomes 4 and 6, respectively. Although Der f 9 was not deposited in IUIS, we obtained a Der p 9-like serine protease sequence located on chromosome 8.

## 4. Conclusions

The genomic data will provide novel insights for the future investigation of the functionally important proteins of disease-related organisms. Our study successfully generated *D. farinae* genome on the chromosome level but did not perform chromosomal structure comparison due to limited mite chromosome information.

## Figures and Tables

**Table 1:** Assembly statistics of the *Dermatophagoides farinae* genome.

	*D. farinae* 2.0 (GenBank no. GCA_000767015.2)		UMICH_USM_1.1 (GenBank no. GCA_002085665.2)		ASM2080927v1 (GenBank no. GCA_020809275.1)		Our work (GenBank no. GCA_024713945.1)	
	Scaffold	Contig	Scaffold	Contig	Scaffold	Contig	Scaffold	Contig
Total number	10	108	1704	1720	10	10	21	22
Total length (bp)	60394945	60120219	91891712	91891214	58776842	58776842	62428314	62428214
Gap number (bp)	274781	0	589	0	0	0	100	0
N50 length (bp)	8981490	2293841	192501	188869	9265486	9265486	7117990	5241854
N90 length (bp)	4366617	606759	20380	19337	3602211	3602211	3348946	3348946
Maximum length (bp)	16911843	3995946	1740722	1740722	13798924	13798924	9172953	9172953
Minimum length (bp)	331559	202	706	706	613882	613882	89727	89727
GC content (%)	30.58	30.58	30.49	30.49	30.38	30.38	30.57	30.57
BUSCO (v5.3.2, arachnida_odb10)	C: 91.4% (S: 89.4%, D: 2.0%), F: 4.0%, M: 4.6%, *n*: 2934		C: 92.4% (S: 72.7%, D: 19.7%), F: 3.5%, M: 4.1%, *n*: 2934		C: 91.7% (S: 90.4%, D: 1.3%), F: 3.7%, M: 4.6%, *n*: 2934		C: 92.1% (S: 90.9%, D: 1.2%), F: 3.6%, M: 4.3%, *n*: 2934	
Sequencing technology	Oxford Nanopore GridION; PacBio Sequel		PacBio; Illumina HiSeq		Nanopore		PacBio; Illumina HiSeq; Hi-C	
Assembly method	Flye v. 2.6		HGAP3 v. 2013		WTDBG v. 1.2.8		FALCON v. 0.3.0; WTDBG2 v. 2.5; Flye v. 2.0	
Genome coverage	431.0x		100.0x		396.0x		265.0x	
Assembly level	Scaffold		Scaffold		Contig		Chromosome	
Assembly release date	Feb 2022		Apr 2017		Nov 2021		Aug 2022	

C: complete BUSCOs; S: complete and single-copy; D: complete and duplicated BUSCOs; F: fragmented BUSCOs; M: missing BUSCOs.

**Table 2:** Transposable elements and repeat sequence statistics.

Sample elements	Number of elements	Length (bp)	Percentage of genome (%)
DNA transposons	736	55004	0.09
LTR	8	370	0.00
Low_complexity	14785	680668	1.16
Simple_repeat	182376	6715274	11.41
Unknown	2083	203508	0.35
srpRNA	1	268	0.00
Total			13.01

LINE: long interspersed unclear elements; LTR: long terminal repeats; SINE: short interspersed nuclear elements; RC: rolling circle; srp RNA: signal recognition particle RNA; rRNA: ribosomal RNA.

**Table 3:** General statistics of the functional annotation.

Database	Number	Percent (%)
InterPro	9524	88.94
Swissprot	6487	60.58
NR	9853	92.01
Pfam	7612	71.08
KEGG	5000	46.69
GO	5930	55.37
eggnog	7853	73.33
Annotated	10342	96.57
Unannotated	367	3.43
Total	10709	—

**Table 4:** The allergens annotated in our genome analysis.

Allergen groups	Deposited in IUIS		Our work gene_ID	Percent identity on nucleotide level (%)
	GenBank nucleotide no.	GenBank protein no.		
Der f 1.0101	AB034946.1	BAC53948.1	Def02G00867	100.00
Der f 2.0102	D10448.1	BAA01240.1	Def01G00220	91.10
Der f 3.0101	D63858.1	BAA09920.1	Def04G01308	98.07
Der f 4.0101	KJ400030.1	AHX03180.1	Def03G00697	96.38
Der f 5.0101	MK814120.1	ABO84970.1	Def04G00810	100
Der f 6.0101	AF125187.1	AAF28423.1	Def06G00935	89.64
Der f 7.0101	S80655.1	AAB35977.1	Def06G00758	90.61
Der f 8.0101	KC305499.1	AGC56215.1	Def05G00166	100
Der f 9.0101	AY211952.1	AAP57077.1	Def08G00480	83.98
Der f 10.0101	D17682.1	BAA04557.1	Def06G00763	93.14
Der f 11.0101	AF352244.1	AAK39511.1	Def03G00266	99.71
Der f 12			Not found	
Der f 13.0101	AY283293.1	AAP35078.1	Def01G00109	100.00
Der f 14.0101	D17686.1	BAA04558.1	Def10G00386	99.43
Der f 15.0101	AF178772.1	AAD52672.1	Def08G00586	100
Der f 16.0101	AF465625.1	AAM64112.1	Def07G00482	100
Der f 17			Not found	
Der f 18.0101	AY093656.1	AAM19082.1	Def05G00671	100
Der f 19			Not found	
Der f 20.0201	EU106619.1	ABU97470.1	Def03G00930	100
Der f 21.0101	KF732965.1	AHC94806.1	Def04G00811	100
Der f 22.0101	DQ643992.1	ABG35122.1	Def06G00004	99.57
Der f 23.0101	KU166910.1	ALU66112.1	Def07G00351	86.36
Der f 24.0101	KC669700	AGI78542	Def03G00188	100
Der f 25.0201	KM010004.1	AIO08860.1	Def01G01174	100
Der f 26.0101	KM009996.1	AIO08852.1	Def03G00168	100
Der f 27.0101	KM009995.1	AIO08851.1	Def04G00246	97.46
Der f 28.0101	KC305502.1	AGC56218.1	Def04G00961	85.79
Der f 29.0101	AY283280.1	AAP35065.1	Def07G00038	99.84
Der f 30.0101	KC305503.1	AGC56219.1	Def01G00231	83.33
Der f 31.0101	KM010014.1	AIO08870.1	Def01G01132	92.57
Der f 32.0101	KM009993.1	AIO08849.1	Def07G00498	100.00
Der f 33.0101	KM010005.1	AIO08861.1	Def04G00806	92.22
Der f 34.0101	LC120618.1	BAV90601.1	Def07G00095	99.24
Der f 35.0101	LC175222.1	BAX34757.1	Def01G00988	100.00
Der f 36.0101	KY465506.1	ATI08931.1	Def08G00335	99.56
Der f 37.0101	MK419030.1	QBF67839.1	Def03G00592	92.00
Der f 38.0101	MN937441	QHQ72282.1	Def03G00068	84.00
Der f 39.0101	MK419032	QBF67841.1	Def09G00137	100

Note: the percent identity was computed by tblastn.

## Data Availability

Genomic sequences and Hi-C data are deposited in NCBI Sequence Read Archive under the BioProject PRJNA756521 and accession number JAMLFS000000000.1. The mitochondrial genome of D. farinae is deposited in NCBI under the accession number OR197578.

## Abbreviations

PacBio: Pacific Bioscience
Hi-C: High-throughput/resolution chromosome conformation capture
BUSCOs: Benchmarking universal single-copy orthologs
WHO: World Health Organization
IUIS: International Union of Immunological Societies
NCBI: National Center for Biotechnology Information
NR: Nonredundant
Der f: *Dermatophagoides farinae.*
